# Impact of universal drug susceptibility testing and effective management of multidrug-resistant tuberculosis in Taiwan

**DOI:** 10.1371/journal.pone.0214792

**Published:** 2019-04-02

**Authors:** Pin-Hui Lee, Pei-Chun Chan, Yen-Ting Peng, Po-Wei Chu, Mei-Hua Wu, Ruwen Jou, Ming-Chih Yu, Chou-Jui Lin, Yi-Wen Huang, Shun-Tien Chien, Jen-Jyh Lee, Chen-Yuan Chiang

**Affiliations:** 1 Taiwan Centers for Disease Control, Taipei, Taiwan; 2 Institute of Epidemiology and Preventive Medicine, College of Public Health, National Taiwan University, Taipei, Taiwan; 3 Division of Pulmonary Medicine, Department of Internal Medicine, Wanfang Hospital, Taipei Medical University, Taipei, Taiwan; 4 School of Respiratory Therapy, College of Medicine, Taipei Medical University, Taipei, Taiwan; 5 Division of Pulmonary Medicine, Department of Internal Medicine, School of Medicine, College of Medicine, Taipei Medical University, Taipei, Taiwan; 6 Pulmonary and Critical Care Unit, Taoyuan General Hospital, Ministry of Health and Welfare, Taoyuan, Taiwan; 7 Pulmonary and Critical Care Unit, Changhua Hospital, Ministry of Health and Welfare, Changhua, Taiwan; 8 Pulmonary and Critical Care Unit, Chest Hospital, Ministry of Health and Welfare, Tainan, Taiwan; 9 Department of Internal Medicine, Buddhist Tzu Chi General Hospital, Tzu Chi University, Hualien, Taiwan; 10 International Union Against Tuberculosis and Lung Disease, Paris, France; Jamia Hamdard, INDIA

## Abstract

**Background:**

The treatment outcomes of multidrug-resistant tuberculosis (MDR-TB) patients in the 1990s in Taiwan was not satisfactory. To strengthen programmatic management of drug-resistant tuberculosis (PMDT), Taiwan MDR-TB Consortium (TMTC) was established in 2007. We assess the performance and epidemiologic impact of TMTC.

**Methodology/Principle findings:**

We analyzed the trends of proportion of TB cases with drug susceptibility testing, enrollment of MDR-TB patients into TMTC and outcomes of treatment of all MDR-TB patients in Taiwan from 2007–2016. We computed the trends of both incidence and prevalence of MDR-TB from 2007–2016. We assessed the trends of MDR-TB among both new and recurrent TB cases. The proportion of TB cases with drug susceptibility testing results increased from 24.2% in 2007 to 97.9% in 2016. Of the 1,452 MDR-TB patients who were eligible for TMTC care, 1,197 (82.4%) were enrolled in TMTC, in whom 82.9% had treatment success. MDR-TB incidence was 9.0 cases per million in 2007, which declined to 4.6 cases per million in 2016 (p<0.0001). MDR-TB prevalence decreased from 19.4 cases per million in 2007 to 8.4 cases per million in 2016 (p<0.0001). The proportion of MDR-TB among new TB cases decreased from 1.4% in 2010 to 1.0% in 2016 (p = 0.039); and that among recurrent TB cases from 9.0% in 2010 to 1.8% in 2016 (p<0.0001).

**Conclusions:**

We concluded that effective PMDT have had a significant impact on the epidemic of drug-resistant TB in Taiwan.

## Introduction

Multidrug-resistant tuberculosis (MDR-TB) remains a public health crisis. In 2017 globally, there were an estimated 558,000 incident tuberculosis (TB) cases with resistance to rifampicin (RR-TB), of which 460,000 were MDR-TB [1]. Only 25% of the 558,000 estimated MDR/RR-TB incident cases were on treatment. Furthermore, treatment outcomes of MDR-TB patients have been unsatisfactory with a treatment success proportion of 55% globally [1].

In the 1990s in Taiwan, 51.2% of newly diagnosed MDR-TB patients were cured and 29.1% were lost to follow-up [2]. In 2007, a government- organized hospital-based management program for MDR-TB patients, Taiwan MDR-TB Consortium (TMTC), was established [3, 4]. The diagnosis and treatment service of TB was provided at no cost to patients through the National Health Insurance program, supplemented by additional funding from the Taiwan Centers for Disease Control (TCDC). The anti-TB drugs used in the treatment of MDR-/RR-TB and extensively drug resistant TB (XDR-TB) were centrally procured by the TCDC. The treatment was individualized for a total duration of 18–24 months, by taking treatment history and results of drug susceptibility testing (DST) into account. The use of bedaquiline may go beyond 6 months as needed. Delamanid was procured for the management of difficult MDR-TB cases in late 2016. A few mobile teams were organized for delivering community-based directly observed therapy (DOT) consistently throughout the whole treatment course. Supportive face-to-face DOT was strictly provided for at least 5 days per week. Psychosocial and financial support was provided to address emotional stress and financial hardship of MDR-TB patients and their families. Previous studies have reported that treatment success among MDR-TB patients in Taiwan increased significantly from 61% in the pre-TMTC era to more than 80% in the TMTC era [3, 4]. Whether an effective programmatic management of drug-resistant TB (PMDT) has an impact on the epidemic of DR-TB has rarely been investigated. Therefore, we assessed the performance and impact of TMTC from 2007 to 2016.

## Materials and methods

### Setting

TB had been a notifiable disease in Taiwan. The notification of all forms of TB in Taiwan was 63.2 per 100,000 population in 2007, which decreased to 43.9 per 100,000 population in 2016 [5, 6]. The completeness of notification of TB had been 96.3% [7]. Notification of MDR-TB had been compulsory since 2007 even if patients had been notified as a TB case previously. Diagnosis and treatment of TB may take place at any health care facility at no cost to patients. A laboratory network with regular external quality assurance of smear microscopy and proficiency testing of DST had been established [8–10]. Patient-centered service of DOT had been provided to smear positive pulmonary TB patients since April 2006, and expanded to all TB patients in 2008 [11]. Treatment outcomes of TB patients registered in 2015 were: 73.0% treatment success, 20.0% died, 0.6% treatment failed, and 6.3% lost to follow-up or outcome-not-evaluated [6]. The high case fatality was because the majority of TB patients were elderly (more than 50% TB cases aged 65 years or more) [6]. Recording of results of DST of first-line anti-TB drugs in the national TB registry began in December 2006 [12]. Clinical audit of anti-TB medications was conducted in 2005 and measures to ensure consistent dosing of anti-TB drugs began in 2007 [13, 14].

### MDR-TB program

In May 2007, the TCDC collaborated with five designated medical care teams to establish TMTC to provide patient-centered treatment service to MDR-TB patients [3, 4]. Incident pulmonary TB with resistance to both isoniazid and rifampicin were eligible for enrollment of TMTC care. Since May 2009, GenoType MTBDR*plus* line probe assay (LPA) were performed among smear positive pulmonary TB patients at risk of drug-resistant TB [15]. Patients with MDR-TB detected by LPA confirmed by a repeat molecular test were also eligible for enrollment to TMTC. Individualized treatment regimens in line with WHO guidelines were used for the treatment of MDR-TB [16]. Patients who were treated by hospitals not part of TMTC were managed under national health insurance and DOT services were provided by the outreach workers of public health centers. The direct medical cost was largely paid by national health insurance. Although they were not treated in TMTC hospitals, clinical issues such as modification of treatment regimens or management of adverse events were discussed as needed in quarterly meetings of TMTC organized by TCDC. The public health nurses of health centers were responsible for assessment of outcomes of treatment of these patients. Patients with chronic infectious TB with no effective treatments available prior to 2015 were reassessed by TB expert committees for possible management after bedaquiline was procured by TCDC in 2015.

To assess the performance of TMTC, we investigated the proportion and timeliness of MDR-TB patient enrollment into TMTC, and the treatment outcomes of patients enrolled in TMTC. To assess the epidemiologic impact of PMDT, we analyzed the trends of the incidence of MDR-TB, the prevalence of MDR-TB, the prevalence of chronic infectious TB cases, and the proportion of MDR-TB among new and recurrent TB cases.

### Detection and enrollment of MDR-TB

Baseline DST of incident culture-positive TB patients notified were collected from the national TB registry from January 1, 2007 to December 31, 2016. We calculated the proportion of TB patients notified with DST results and the proportion of MDR-TB among new and recurrent TB patients by calendar year (S1 Fig). All incident pulmonary TB patients whose clinical specimens were collected from January 1, 2007 to December 31, 2016 were included to assess the annual incidence of MDR-TB. The demographic and clinical characteristics of MDR-TB patients were obtained from the national TB registry. The information collected included age, sex, history of anti-TB treatment, baseline sputum AFB smear results, baseline DST and treatment outcomes.

### Performance and impact of PMDT

We calculated the proportion of MDR-TB patients enrolled into TMTC among all eligible patients and measured the interval between collection of sputum which confirmed the diagnosis of MDR-TB and enrollment into TMTC by year from 2007 to 2016 (S2 Fig). We analyzed the 36-month treatment outcomes of all MDR-TB patients who were diagnosed between 1 January 2007 and 31 August 2014 regardless of TMTC enrollment. (S2 Fig) The treatment outcomes were classified according to WHO’s treatment outcome definitions [17]. Outcomes of patients who had drugs changed because of adverse reactions were not categorized as treatment failure. We assessed the number of chronic infectious TB patients under management on June 30 annually from 2007 to 2016. The DST results among prevalent chronic infectious patients were also analyzed.

### Statistical analysis

The trend of proportion of patients with DST was analyzed using the chi-square trend test. The trend of mean intervals between diagnosis and TMTC enrollment was analyzed using linear regression models. Poisson regression models were constructed to analyze the trend of MDR-TB incidence and prevalence, and the trend of chronic infectious TB prevalence. We analyzed the trend of proportion of MDR-TB among all TB cases from 2010 to 2016, because the proportion of patients with DST was relatively low before 2010. The trends of proportion of MDR-TB among new and recurrent TB cases were analyzed using the chi-square trend test. All analyses were conducted using SAS software version 9.3 (SAS Institute, Cary, North Carolina).

### Ethics

This study was approved by the ethics committee of the Taiwan Centers for Disease Control (IRB No. 107205).

## Results

From 2007 to 2016, a total of 130,631 incident culture positive TB cases (new and recurrent) were notified, among which, 74,141 had baseline DST results available in the national TB registry. The proportion of patients with DST results in the national TB registry was 24.2% (24.1% among new and 26.7% among recurrent cases) in 2007, reached 93.7% (93.7% among new and 93.0% among recurrent cases) in 2010, and further increased to 97.9% (97.9% among new and 100.0% among recurrent cases) in 2016 (Table 1).

**Table 1:** Number and proportion of culture positive tuberculosis cases with baseline drug susceptibility testing results, 2007–2016.

year	2007	2008	2009	2010	2011	2012	2013	2014	2015	2016
Number of total TB cases	15,599	15,269	14,115	13,991	13,351	12,796	11,960	11,744	11,125	10,681
Number of culture positive cases	10,177	10,494	9,921	10,253	10,171	9,658	9,124	9,093	8,604	8,406
Number with DST*	2,464	3,196	5,206	9,607	9,885	9,374	8,854	8,847	8,477	8,231
proportion with DST	24.2%	30.5%	52.5%	93.7%	97.2%	97.1%	97.0%	97.3%	98.5%	97.9%
Case classification										
New TB cases										
Number of total cases	14,825	14,596	13,526	13,383	12,768	12,317	11,512	11,314	10,704	10,500
Number of culture positive cases	9728	10083	9567	9870	9806	9365	8842	8837	8358	8294
Number with DST	2,344	3,037	4,995	9,251	9,532	9,093	8,583	8,603	8,234	8,119
proportion with DST	24.1%	30.1%	52.2%	93.7%	97.2%	97.1%	97.1%	97.4%	98.5%	97.9%
Recurrent TB cases										
Number of total cases	774	673	589	608	583	479	448	430	421	181
Number of culture positive cases	449	411	354	383	365	293	282	256	246	112
Number with DST	120	159	211	356	353	281	271	244	243	112
proportion with DST	26.7%	38.7%	59.6%	93.0%	96.7%	95.9%	96.1%	95.3%	98.8%	100.0%

*DST, drug susceptibility testing.

A total of 1,530 MDR-TB patients with 1,535 episodes were identified from 2007 to 2016 in the national TB registry. 1,452 (94.9%) MDR-TB patients were eligible for TMTC care, in whom 1,197 (82.4%) were enrolled in TMTC (S3 Fig). The mean interval between collection of clinical specimens that were found to be MDR-TB and enrollment into TMTC was 5.3 months in 2007, which decreased to 2.2 months in 2016 (S1 Fig, p for trend = 0.0008). The median age of MDR-TB patients enrolled in TMTC was 54.0 years (IQR: 41.9–67.0), which was younger than those who were not enrolled in TMTC (median: 65.7 years, IQR: 48.5–78.4). Compared to those who were enrolled in TMTC, a significantly higher proportion of those without TMTC care were new TB patients (56.8% vs. 74.1%, p <0.0001) and had XDR-TB (1.8% vs. 3.9%, p = 0.045) (Table 2).

**Table 2:** Demographic and clinical characteristics of incident MDRTB patients, 2007–2016.

	TMTC* (n = 1,197)		Non-TMTC (n = 255)		Total (n = 1,452)
	n	%	n	%	n
Gender					
male	875	73.1%	193	75.7%	1,068
female	322	26.9%	62	24.3%	384
Age group (year)					
<25	65	5.4%	4	1.6%	69
25–44	298	24.9%	43	16.9%	341
45–64	515	43.0%	77	30.2%	592
≧65	319	26.7%	131	51.4%	450
Case classification					
New	680	56.8%	189	74.1%	869
Previously treated					
with first-line drugs	310	25.9%	36	14.1%	346
with second-line drugs	79	6.6%	16	6.3%	95
Others	128	10.7%	14	5.5%	142
Susceptibility of FQ and SLI^†^					
FQ-S, SLI-S	853	71.3%	131	51.4%	984
FQ-R, SLI-S	130	10.9%	26	10.2%	156
FQ-S, SLI-R	61	5.1%	19	7.5%	80
FQ-R, SLI-R	21	1.8%	10	3.9%	31
missing	132	11.0%	69	27.1%	201
AFB smear					
Positive	716	59.8%	141	55.3%	857
Negative	480	40.1%	102	40.0%	582
Unknown	1	0.1%	12	4.7%	13

* TMTC, Taiwan Multidrug-resistant Tuberculosis Consortium.

†FQ, fluoroquinolone; SLI, second line injectables; S, susceptible; R, resistance.

Treatment outcomes at 36-months among MDR-TB patients who were diagnosed between 1 January 2007 and 31 August 2014 are shown in Table 3. The proportion of treatment success among MDR-TB patients enrolled in TMTC was 82.9%, much higher than that among those not enrolled in TMTC (28.7%). A high proportion of patients not enrolled in TMTC died; the majority died within 2 months of clinical specimens collection (41.6%).

**Table 3:** Treatment outcomes of patients with multidrug-resistant tuberculosis.

	Patients enrolled in TMTC* N = 986	Patients not enrolled in TMTC N = 216
	Number (percentage)	
Treatment success	817 (82.9)	62 (28.7)
Death	126 (12.8)	126 (58.3)
Treatment failure	8 (0.8)	2 (0.9)
Lost to follow-up	16 (1.6)	6 (2.8)
Outcome not evaluated	19 (1.9)	20 (9.3)

* TMTC, Taiwan Multidrug-resistant Tuberculosis Consortium.

The incidence of MDR-TB decreased from 9.0/1,000,000 in 2007 to 4.6/1,000,000 in 2016 (49% reduction, p for trend <0.0001; Table 4). The gender- and age-specific incidence of MDR-TB also decreased significantly from 2007 to 2016 (Table 4). Of the 205 incident MDR-TB cases in 2007, 82 (40%) were new TB cases and 123 (60%) were previously treated. The number of incident MDR-TB cases decreased to 107 in 2016 (48% reduction), among which, 70 (65%) were new cases (15% reduction compared to 2007) and 37 (35%) were previously treated TB cases (70% reduction compared to 2007).

**Table 4:** Number and incidence (per million population) of MDR-TB from 2007 to 2016, by case classification, sex and age groups.

Year	2007	2008	2009	2010	2011	2012	2013	2014	2015	2016
Total MDR-TB cases, n = 1,529	205	220	175	159	147	148	123	120	125	107
New, n = 932	82	127	97	95	96	104	83	88	90	70
Previously treated, n = 597	123	93	78	64	51	44	40	32	35	37
Incidence rate	9.0	9.6	7.6	6.9	6.3	6.4	5.3	5.1	5.3	4.6
Incidence rate by gender										
Male*	13.4	13.0	10.5	9.2	9.2	9.4	8.0	7.1	7.9	7.3
Female*	4.4	6.1	4.6	4.5	3.5	3.3	2.6	3.2	2.8	1.9
Incidence rate by age groups (year)										
Age <25*	2.0	2.2	1.7	1.3	1.2	0.9	1.1	0.8	1.1	0.3
Age 25–44^†^	8.0	7.6	5.9	6.2	3.9	5.5	3.8	2.7	4.2	2.5
Age 45–64^†^	15.5	15.6	11.2	8.3	10.0	9.3	8.7	7.2	6.6	5.0
Age ≥65^‡^	18.6	23.7	21.0	21.0	18.4	15.7	11.4	16.7	14.3	17.2

* p for trend = 0.0003

^**†**^ p for trend <0.0001

^‡^p for trend = 0.007

**Fig 1:**
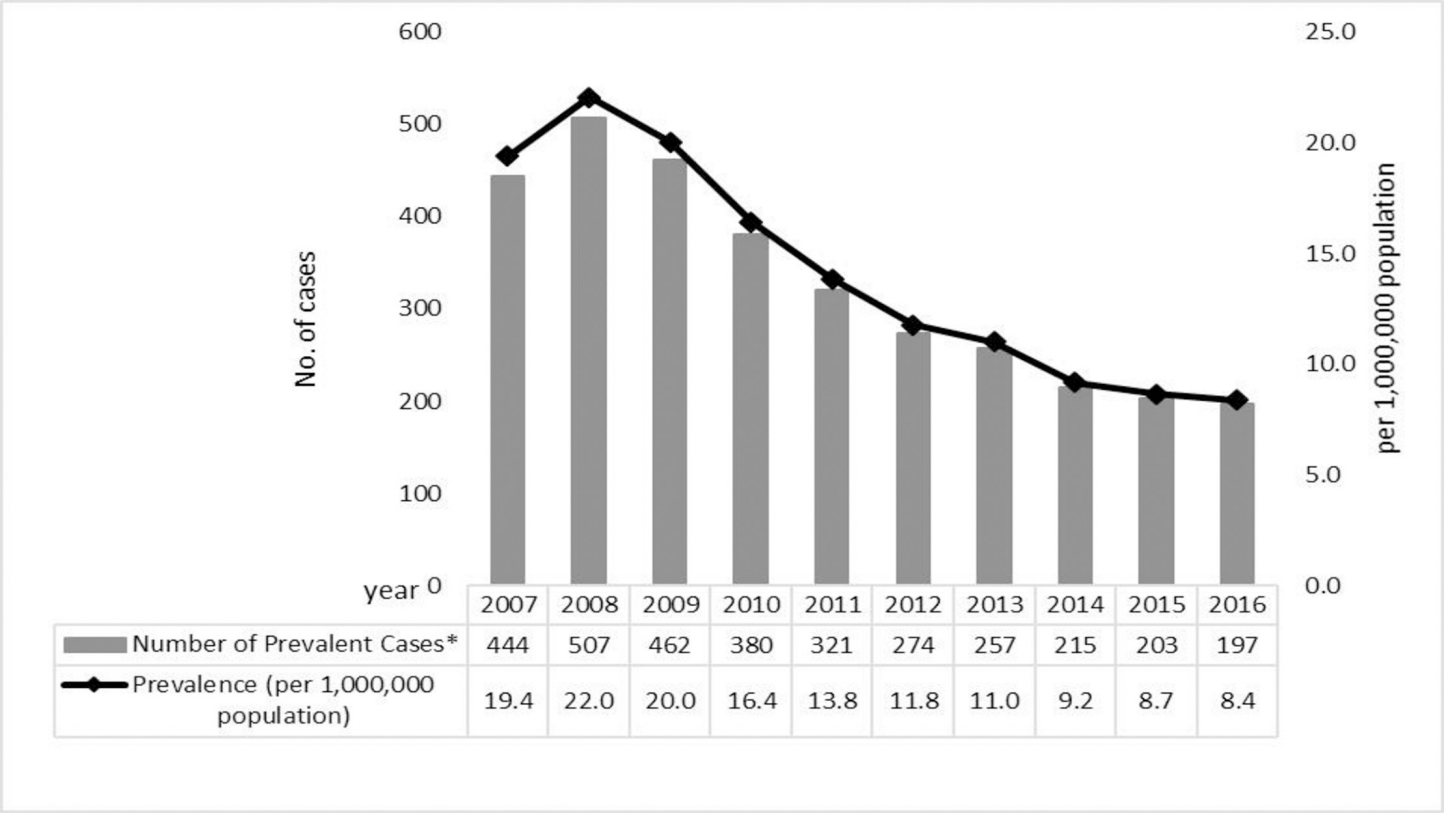
Number and rate (per million population) of prevalent MDR-TB cases on June 30 each year in Taiwan, 2007–2016.

The prevalence of MDR-TB patients increased from 19.5/1,000,000 in 2007 to 22.1/1,000,000 in 2008, and decreased to 8.4/1,000,000 in 2016 (57% reduction compared to 2007, p for trend < 0.0001; Fig 1).

The prevalence of chronic infectious TB cases peaked at 1.3/1,000,000 (34 cases) in 2009, which decreased to 0.3/1,000,000 (7 cases) in 2016 (77% reduction) (S4 Fig). Four patients who had previously been diagnosed with chronic infectious TB received bedaquiline-containing regimens in 2015. By the end of 2016, three completed treatment and one remained under TMTC care.

**Fig 2:**
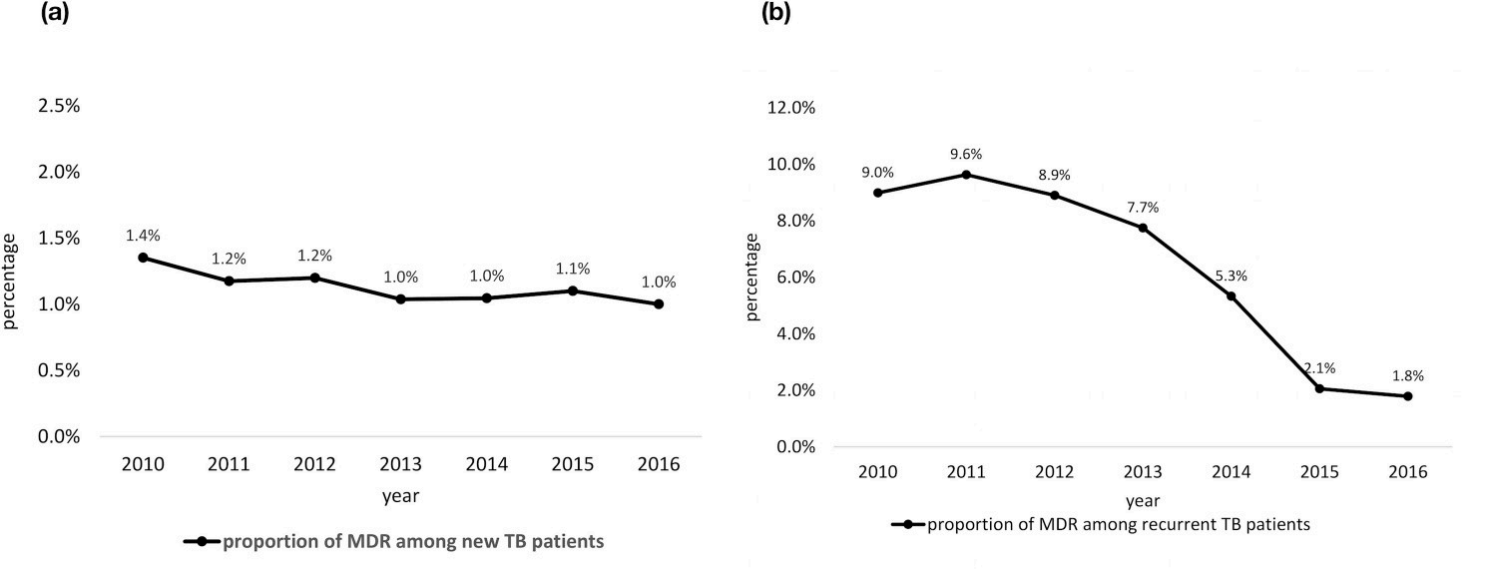
(a) Proportion of culture positive new tuberculosis patients who had MDR-TB in Taiwan, 2010–2016. (b) Proportion of culture positive recurrent tuberculosis patients who had MDR-TB in Taiwan, 2010–2016.

The proportion of MDR-TB among new TB cases was 1.4% in 2010, which decreased to 1.0% in 2016 (29% reduction, p for trend = 0.039). The proportion of MDR-TB among recurrent TB cases was 9.0% in 2010, which decreased to 1.8% in 2016 (80% reduction, p for trend <0.0001; Fig 2).

## Discussion

In our study, the proportion of culture-positive TB patients with DST results in the national TB registry reached 90% in 2010. A high proportion of MDR-TB patients eligible for TMTC were enrolled and the interval between diagnosis and enrollment into TMTC shortened progressively. The proportion of MDR-TB patients with treatment success maintained above 80%. Consequently, the incidence and prevalence of MDR-TB cases and the prevalence of chronic infectious TB cases in Taiwan decreased significantly. Furthermore, the proportion of MDR-TB among new and recurrent TB patients decreased substantially, because transmission of MDR-TB had been mitigated by an effective PMDT and a sound DOT program had reduced the risk of acquired resistance in the treatment of TB.

The proportion of TB patients with DST results in the national TB registry was relatively low in 2007, because of incomplete upload of DST results to the TB registry. As TB laboratory services were fully covered by the National Health Insurance (NHI), most culture positive TB patients in Taiwan had DST. However, such information was not routinely collected before December 2006. Furthermore, since 2007, second line drugs for the treatment of complicated TB or DR-TB patients were mainly supplied by TCDC and submitting results of DST was required when requesting drugs; this enabled TCDC to actively collect DST information of TB patients requiring the use of second line drugs.

The increased proportion of MDR-TB patients enrolled into TMTC and decreased interval between diagnosis and TMTC enrollment were the results of enhanced collaboration between hospitals which referred MDR-TB patients to TMTC, public health staff and TMTC. Increased use of rapid molecular diagnostics in the detection of MDR-TB may have helped in reducing the interval of diagnosis and enrollment. Only 17.6% of patients declined treatment at TMTC for a variety of reasons. However, the main reason of non-enrollment was patient death shortly after sputum collection.

Outcomes of MDR-TB patients under TMTC care during 2007–2012 have been published, reporting that more than 82% of MDR-TB patients had treatment success [4]. Our analysis found that TMTC has been able to maintain high proportion of treatment success. Among those who were not enrolled in TMTC, 87% also became epidemiologically neutral because they were either cured or died. Furthermore, recurrent MDR-TB among those who have been successfully treated was low (3% during a median follow-up of 4.8 years) [18].

The decreasing incidence of MDR-TB was most prominent among those who had been previously treated. Patients with previously treated TB accounted for 60% of MDR-TB patients enrolled in 2007, which decreased to 35% in 2016. The decrease of MDR-TB among previously treated TB cases was higher than that among new TB cases (15% decrease). The decrease of MDR-TB among previously treated TB patients is in part due to the remarkable reduction of recurrent TB cases following the implementation of DOT. TB patients enrolled in DOT were more likely to be treated successfully, and the risk of acquiring isoniazid or rifampicin resistance reduced [19–21]. The effective DOT program also contributed to a significant reduction of the proportion of MDR-TB among recurrent TB cases from 9.0% in 2010 to 1.8% in 2016.

The prevalence of MDR-TB depends on the incidence of MDR-TB and the duration of disease. As the incidence of MDR-TB reduced substantially by 49% during 2007–2016 and the duration of disease was shortened by effective case management, the prevalence of MDR-TB decreased by 57% from 2007 to 2016. Furthermore, effective case management also prevent the generation of new chronic infectious TB cases. As existing chronic infectious cases died over time, and a small fraction of existing chronic infectious cases were cured by bedaquiline and other repurposed drugs, we managed to drastically reduce the prevalence of chronic infectious TB cases by 70% from 2007 to 2016. Consequently, transmission of MDR-TB was averted, resulting in the reduction of the proportion of MDR-TB among new TB cases from 1.4% in 2010 to 1.0% in 2016.

Our study has strengths. First, it is a population-based study that covers the total population in Taiwan. Second, PMDT in Taiwan has been in consistent operation with good collaboration between partners for 10 years, providing us with quality data for this analysis. This study has some limitations. First, we used notification data to assess the incidence of MDR-TB, but there might be gap between incidence and notification if under diagnosis and under reporting of TB is substantial. However, this is unlikely because TB services in Taiwan have been provided completely free to patients and completeness of notification of TB has been very high. Second, we did not assess the number of patients who required changes of two drugs due to adverse reactions and classify such cases as treatment failure, and may have underestimated the proportion of treatment failure [18]. However, this would not have changed the findings of the incidence and prevalence of MDR-TB, nor the proportion of MDR-TB among new and recurrent TB patients.

The TMTC model of care in collaboration with public health sectors has been efficient in the enrollment and effective in the treatment of MDR-TB patients. We conclude that universal DST and effective PMDT have had a significant impact on the epidemic of drug-resistant TB in Taiwan.

## Supporting information

S1 Fig Flow diagram of drug susceptibility testing (DST) among new and recurrent tuberculosis patients. (TIF)

S2 Fig Flow diagram of analysis of the program performance. (TIF)

S3 Fig Proportion and timeliness of eligible MDR-TB patients enrolled into the TMTC care system, 2007–2016. (TIF)

S4 Fig Number and prevalence (per million population) of chronic infectious tuberculosis cases on June 30 each year in Taiwan, 2007–2016. (TIF)
