# Supplementary figures and images for: Impact of universal drug susceptibility testing and effective management of multidrug-resistant tuberculosis in Taiwan
Source: PLoS One. 2019 Apr 2;14(4):e0214792. doi: 10.1371/journal.pone.0214792 (PMC6445419; doi:10.1371/journal.pone.0214792)

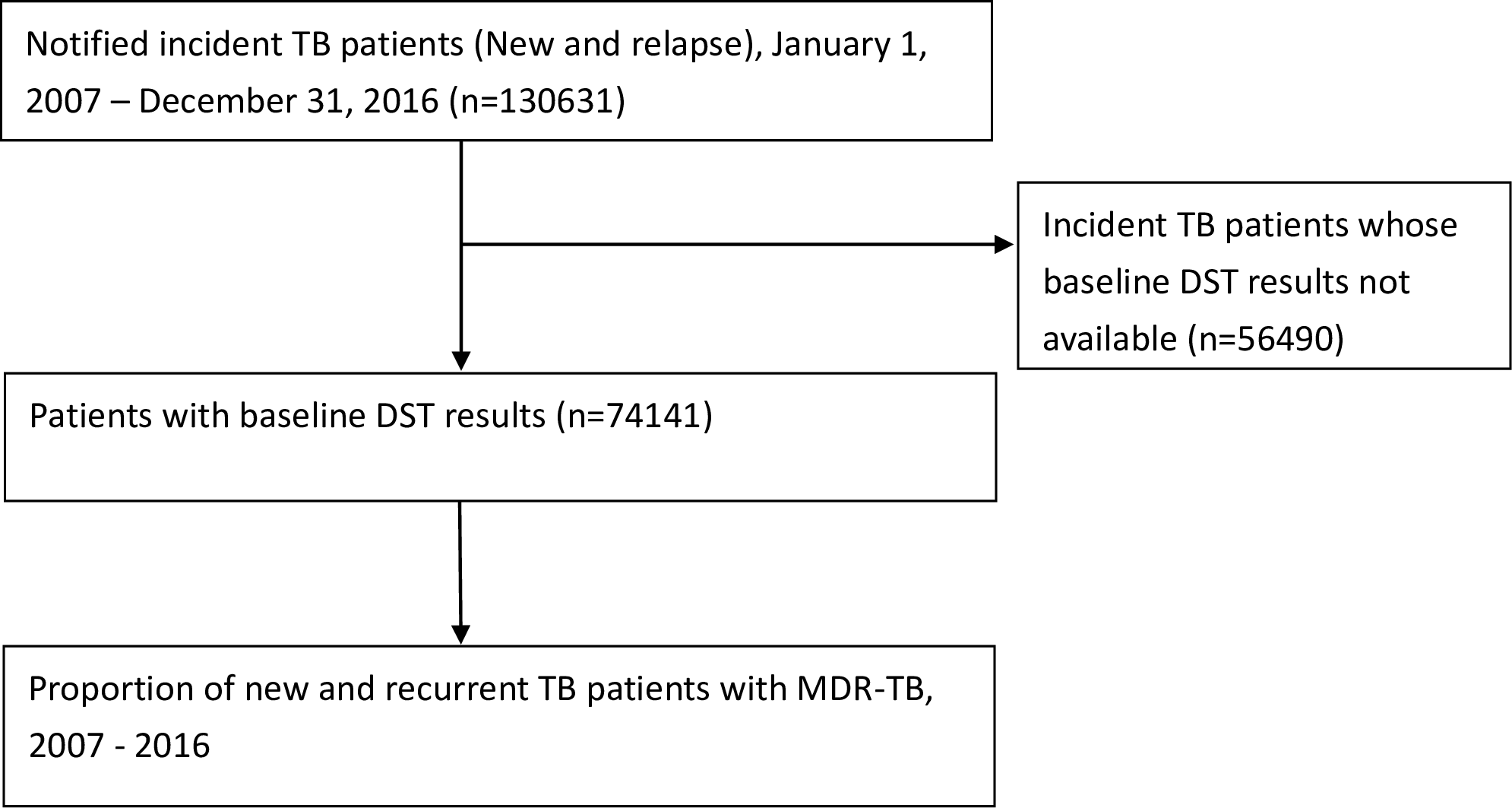

Supplement: S1 Fig — (TIF) [file pone.0214792.s001.tif]

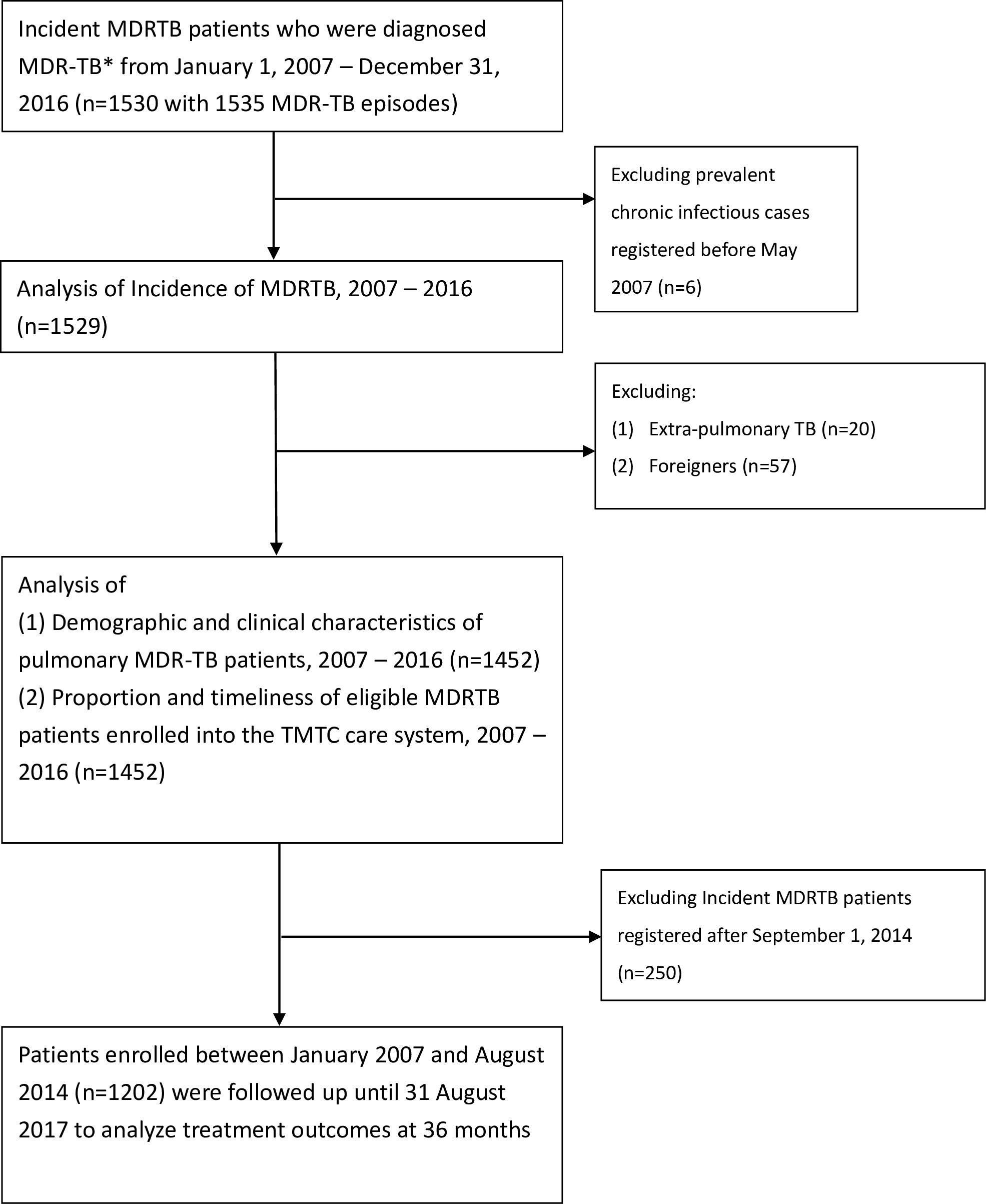

Supplement: S2 Fig — (TIF) [file pone.0214792.s002.tif]

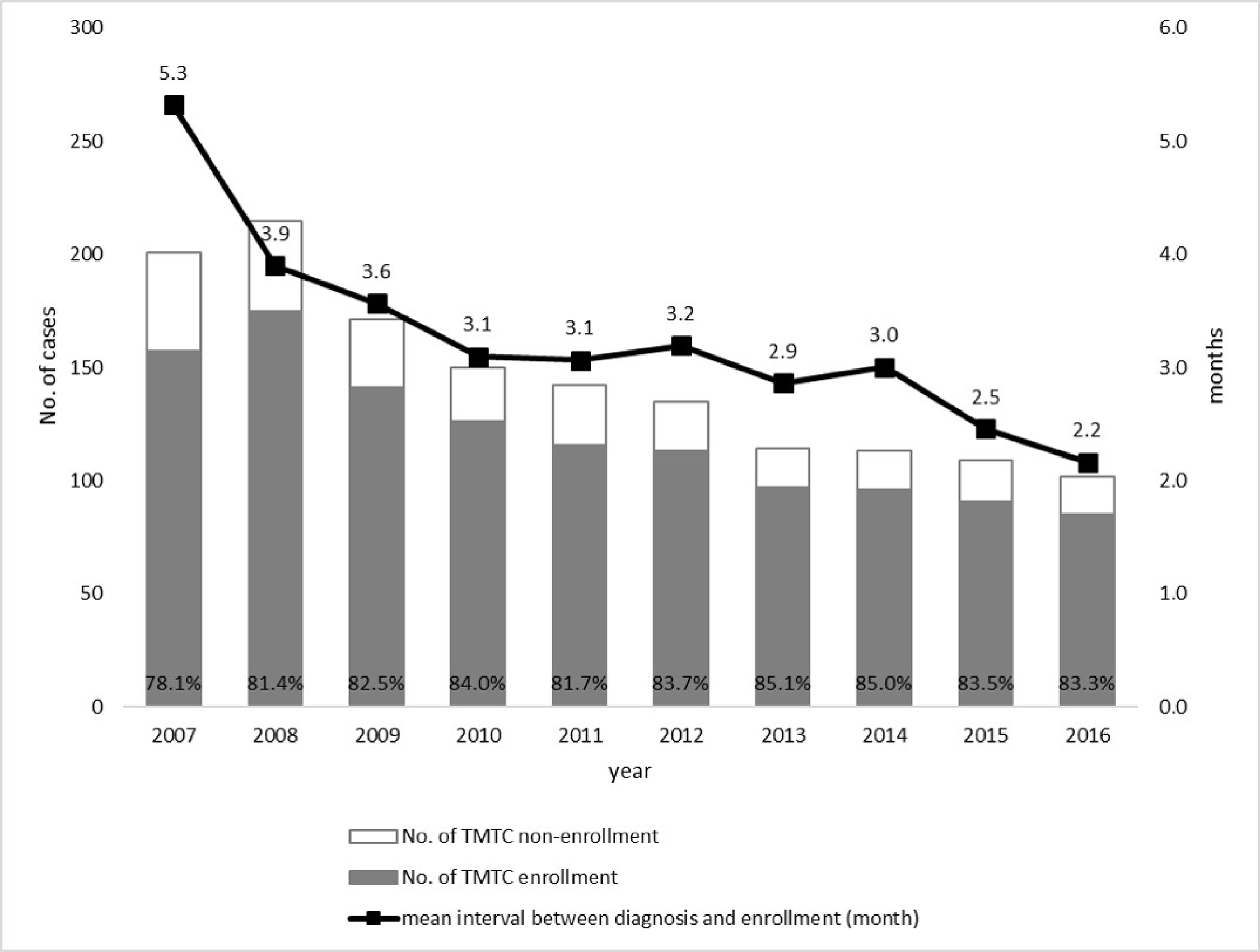

Supplement: S3 Fig — (TIF) [file pone.0214792.s003.tif]

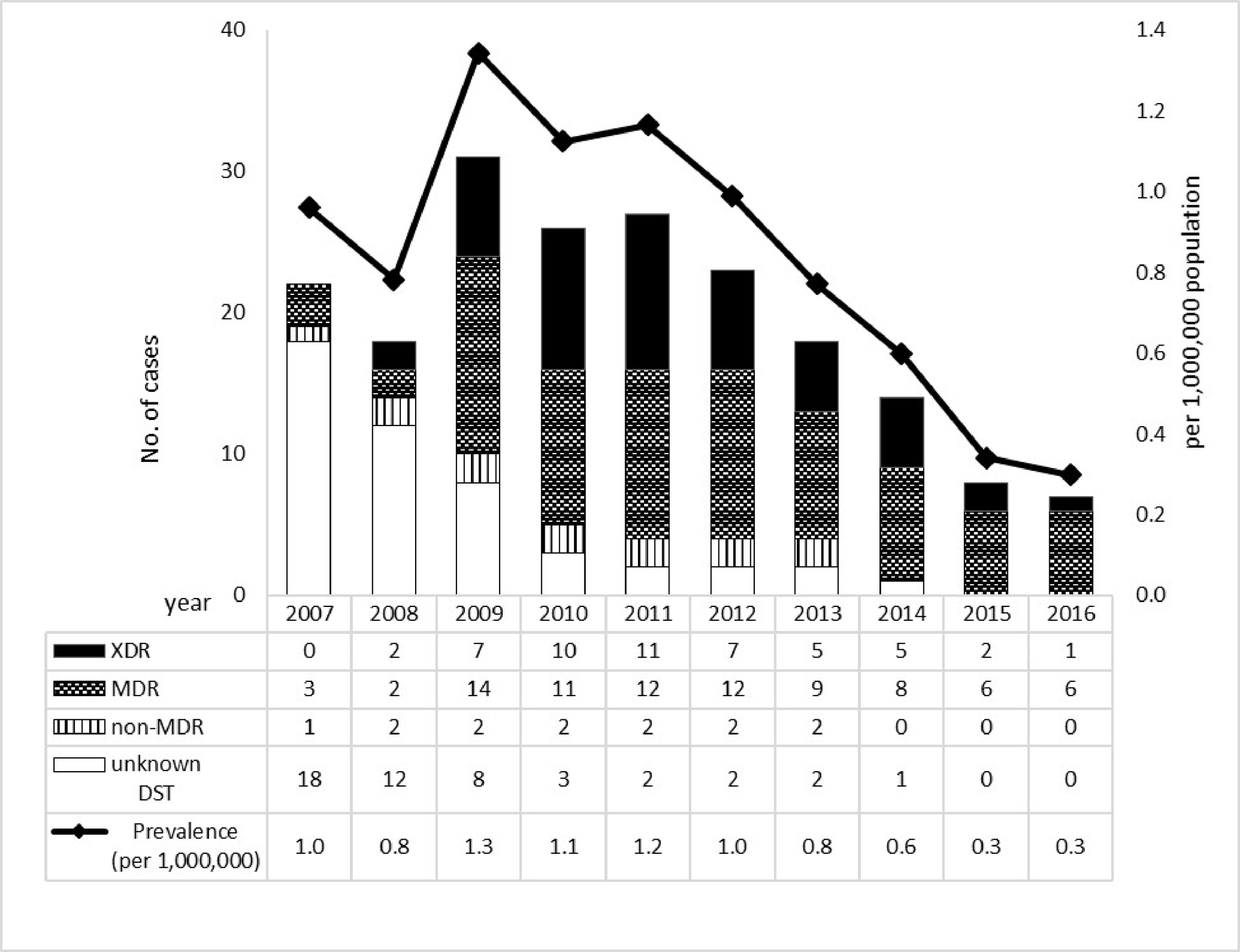

Supplement: S4 Fig — (TIF) [file pone.0214792.s004.tif]
